# Gut Microbiota Species Can Provoke both Inflammatory and Tolerogenic Immune Responses in Human Dendritic Cells Mediated by Retinoic Acid Receptor Alpha Ligation

**DOI:** 10.3389/fimmu.2017.00427

**Published:** 2017-04-18

**Authors:** Krisztian Bene, Zsofia Varga, Viktor O. Petrov, Nadiya Boyko, Eva Rajnavolgyi

**Affiliations:** ^1^Faculty of Medicine, Department of Immunology, University of Debrecen, Debrecen, Hungary; ^2^Faculty of Medicine, R&D Centre of Molecular Microbiology and Mucosal Immunology, Uzhhorod National University, Uzhhorod, Ukraine

**Keywords:** monocyte-derived dentritic cell, gut microbiota, all-*trans* retinoic acid, retinoic acid receptor alpha, interferon regulatory factor 4, T cell, CD1a, CD1d

## Abstract

Dendritic cells are considered as the main coordinators of both mucosal and systemic immune responses, thus playing a determining role in shaping the outcome of effector cell responses. However, it is still uncovered how primary human monocyte-derived DC (moDC) populations drive the polarization of helper T (Th) cells in the presence of commensal bacteria harboring unique immunomodulatory properties. Furthermore, the individual members of the gut microbiota have the potential to modulate the outcome of immune responses and shape the immunogenicity of differentiating moDCs *via* the activation of retinoic acid receptor alpha (RARα). Here, we report that moDCs are able to mediate robust Th1 and Th17 responses upon stimulation by *Escherichia coli Schaedler* or *Morganella morganii*, while the probiotic *Bacillus subtilis* strain limits this effect. Moreover, physiological concentrations of all-*trans* retinoic acid (ATRA) are able to re-program the differentiation of moDCs resulting in altered gene expression profiles of the master transcription factors RARα and interferon regulatory factor 4, and concomitantly regulate the cell surface expression levels of CD1 proteins and also the mucosa-associated CD103 integrin to different directions. It was also demonstrated that the ATRA-conditioned moDCs exhibited enhanced pro-inflammatory cytokine secretion while reduced their co-stimulatory and antigen-presenting capacity thus reducing Th1 and presenting undetectable Th17 type responses against the tested microbiota strains. Importantly, these regulatory circuits could be prevented by the selective inhibition of RARα functionality. These results altogether demonstrate that selected commensal bacterial strains are able to drive strong effector immune responses by moDCs, while in the presence of ATRA, they support the development of both tolerogenic and inflammatory moDC in a RARα-dependent manner.

## Introduction

The development and the metabolic activity of the human immune system critically depend on the amount and the diversity of the human microbiota acquired from the actual tissue microenvironment (1, 2). Upon birth, the human gastrointestinal tract becomes colonized by commensal microbes co-evolved with humans in a symbiotic or at least mutualistic manner together with the immune system (3, 4). The local dendritic cell (DC) network involves a highly heterogeneous population of cells of myeloid and bone marrow origin (5), and in the course of this balancing regulation, moDCs also act as potent organizers of adaptive immunity leading to the maintenance of peripheral tolerance against the gut resident microbes. However, our knowledge about the interplay of molecular interactions during diet involving vitamin A supplementation, and the presence of gut microbiota species in the course of an ongoing human immune system is still limited in both health and diseases.

The uncontrolled disruption of the gut microbiota can be provoked by dysbiosis due to excessive hygiene conditions and/or the presence of antibiotics. This microbial perturbation may play role in the pathogenesis of chronic inflammatory and autoimmune diseases such as inflammatory bowel diseases (IBD), celiac disease, allergy, and metabolic and neurobehavioral diseases. For example, in Crohn’s disease, the ratio of *Proteobacteria* could be increased (6), while the diversity and the fraction of *Firmicutes* in the gut microbiota are decreased (7). Colonization with commensal *Escherichia coli* 083 and *Lactobacillus rhamnosus* strains in early life is able to decrease the incidence of allergies and atopic dermatitis, respectively (8, 9). The various effects of probiotic gut bacteria also may prevent infection by pathogens such as the probiotic *E. coli Nissle* 1917 strain, which is able to inhibit the growth of enteropathogenic *E. coli*, which also may serve as a safe strain in IBD treatment (10–12).

Here, we focus to the underlying mechanisms involved in the recognition and processing of different species of gut commensal and beneficial bacteria and to their ability to polarize helper T (Th) lymphocytes. Considering that the human commensal microbiota is personalized (13) and exhibits high heterogeneity, it also contributes to the development of protective immune responses against pathogens *via* modulating the type and the composition of gut resident effector T cells (13–15). It is well established that pathogenic microbes or pathobionts, including fungal and bacterial species, are able to induce different types of immune responses (16, 17), which are modulated by external and internal signals. However, the means how non-pathogenic gut commensal species contribute to the coordination and fine tuning of immune responses by moDCs is not completely uncovered. In line with this, the primary goal of this study was to characterize a selected set of the normal gut microbiota including *Escherichia coli var. mutabilis* (*E. coli Schaedler*), *Morganella morganii* from *Proteobacteria*, and probiotic *Bacillus subtilis* 090 from *Firmicutes*, all with individual immunogenic and/or modulatory potential during moDC maturation and T-lymphocyte polarization. As it has previously been described, *E. coli Schaedler* and *M. morganii* exert unique stimulatory effects on the developing immune system and are also able to induce oral tolerance in mice (18), while *B. subtilis* is widely used in veterinary practice based on the active constituents of probiotic Monosporyn™ developed at the Uzhhorod National University. Upon interaction with the mucosal immune system, tolerogenic immune responses are raised against commensal and beneficial microbes. However, it is still poorly understood how the special but highly complex and dynamic intestinal milieu impacts the differentiation program of moDCs and the outcome of moDC-mediated immunological processes initiated by normal microbiota members and probiotic bacteria such as *B. subtilis* 090.

The differentiation program of monocytes during moDC generation is initiated by granulocyte-macrophage colony-stimulating factor (GM-CSF) and interleukin (IL)-4 and is regulated by the peroxisome proliferator-activated receptor gamma (PPARγ) (19). PPARγ is known to collaborate with retinoid receptors and acts as a master transcriptional regulator in human moDC differentiation and function (19). In addition, a set of genes encoding proteins related to metabolism, lipid antigen processing and presentation, invariant natural killer T (iNKT) cell activation, and RA synthesis are regulated by PPARγ and overlaps with those regulated by retinoic acid receptor alpha (RARα) (20–23), showing that RARα also serves as a master regulator of moDC functions. In humans, the vitamin A derivate all-*trans* retinoic acid (ATRA) is produced endogenously from retinol by DCs, macrophages, and epithelial and stromal cells (20, 24–27) and binds to RARα and retinoic X receptor alpha (RXRα) with different affinities (28) and enables to follow up the modulatory effects of the retinoid pathways in moDC-mediated immune responses. Besides targeting the highly conserved receptor RARα (29), ATRA also serves as a potential therapeutic drug in anticancer settings (30) and in combinations with other therapeutic agents such as GM-CSF (31) able to promote myelomonocytic differentiation.

We hypothesize that human monocytes migrating from the blood to the intestinal *lamina propria* have access to these special microenvironments, which are conditioned by growth factors and metabolites, including GM-CSF, exogenous and/or endogenous ATRA, and take part in the coordination of immune responses raised against the targeted gut commensal species. Intestinal mononuclear cells express mucosa-associated cell surface molecules such as CX_3_CR1 and/or CD103 (32, 33). The main sources of human intestinal CX_3_CR1^+^ DCs are circulating monocytes, which lose this marker within 24 h (34). In contrast to this event, the CX_3_CR1 chemokine receptor remains expressed on the cell surface of intestinal mononuclear phagocytes and acts directly as an inflammatory and migratory cell population with high phagocytic capacity (34–37), while mucosal CD103^+^ DCs have been described as a dominant migratory population involved in triggering regulatory T cell responses raised against commensal bacteria *via* producing RA (38).

Based on this concept, *in vitro* conditions were designed to analyze the canonical pathways leading to the ATRA-modulated expression of the contributing master transcription factors including retinoid receptors, PPARγ and interferon regulatory factor 4 (IRF4) playing role in moDC differentiation in line with the impact of different, individual commensal bacteria exerted on moDC-mediated inflammation and effector T-lymphocyte priming. In this context, we will follow up the phenotypic changes and the functional activities of moDC populations by monitoring their phagocytic potential, inflammatory nature, and immunogenicity. Taken the unique intestinal microenvironment and the complex interplay of various exogenous effects, we sought to demonstrate how external and internal stimuli derived from the engulfed commensal *E. coli Schaedler, M. morganii*, and the probiotic *B. subtilis* bacteria may impact on the development of effector T-lymphocyte activation and polarization followed up by the production of interferon gamma (IFNγ) and IL-17 cytokines.

## Materials and Methods

### Bacterial Strains and Reagents

The experiments were performed with the commensal bacteria as follows: *E. coli var. mutabilis (Schaedler)* (O83:K24:H31, member of the original Schaedler’s flora), *M. morganii*, and *B. subtilis* 090. *M. morganii* was kindly provided by Michael Potter, National Institutes of Health, strain *E. coli Schaedler* was obtained from Russel Schaedler, USA, and *B. subtilis* 090 was provided by Nadiya Boyko, National University of Uzhhorod, Ukraine. Both commensal gut microbiota strains were received by the R&D Centre for Molecular Microbiology and mucosal immunology from Pennsylvania University in the framework of a research cooperation agreement. ATRA, the selective RARα antagonist BMS-195614 (BMS614), the vehicle dimethyl-sulfoxide (DMSO), and the anti-hβ-actin mAb were from Sigma-Aldrich, Schnelldorf, Germany. The anti-hIRF4 antibody was from Cell Signaling Technology, Inc. (Trask Lane, Danvers, MA, USA).

### Human moDC Cultures

Peripheral blood mononuclear cells (PBMCs) were separated by a standard density gradient centrifugation with Ficoll-Paque Plus (Amersham Biosciences, Uppsala, Sweden). Monocytes were purified from PBMCs by positive selection using immunomagnetic cell separation and anti-CD14 microbeads, according to the manufacturer’s instruction (Miltenyi Biotec, Bergisch Gladbach, Germany). After separation on a VarioMACS magnet, 96–99% of the cells were shown to be CD14^+^ monocytes, as measured by flow cytometry. Isolated monocytes were cultured for 2 days in 12-well tissue culture plates at a density of 5.0 × 10^5^ cells/ml in Gibco’s serum-free AIM-V medium (Thermo Fischer Scientific, Waltham, MA, USA) supplemented with 80 ng/ml GM-CSF (Gentaur Molecular Products, Brussels, Belgium) and 100 ng/ml IL-4 (PeproTech EC, London, UK). The cells were differentiated in the presence or absence of 1 nM ATRA followed by a 75-min incubation period with or without 1 μM BMS614 specific RARα-antagonist at 37°C atmosphere containing 5% CO_2_.

### Bacterial Growth for moDC Activation

Selected gut commensal bacteria were grown in 2% lysogeny broth medium (Serva Electrophoresis GmbH, Heidelberg, Germany) for overnight with shaking at 37°C. Bacterial suspensions were washed with 25 ml sterile phosphate-buffered saline (PBS) three times and OD_600nm_ was measured by spectrophotometry and converted to cell/ml following OD_600nm_ × 2.5 × 10^8^ CFU/ml. Human moDC cultures were activated with the specific toll-like receptor ligand bacterial lipopolysaccharide (LPS) (250 ng/ml ultrapure LPS, InvivoGen, San Diego, CA, USA) and with live commensal bacteria at a non-toxic ratio of 1:0.4 and were co-cultured for another 24 h.

### Phagocytosis Assay

Live bacterial cells were centrifuged at 1,000 × *g* for 5 min and washed three times in 25 ml PBS. Suspensions of bacterial cells were heat inactivated by heating at 65°C for 45 min and were re-suspended in 0.25 M carbonate–bicarbonate buffer (pH 9.0). The heat-killed bacterial cell suspensions (900 μl) were stained with 100 μl fluorescein-isothiocyanate (FITC) used at 5 mg/ml dissolved in DMSO and were rotated overnight at 4°C in dark. FITC-labeled bacteria were washed three times with cold PBS and were co-incubated for 3 h with moDCs at 37 or 4°C at a moDC:bacteria ratio of 1:20. moDCs positive for FITC-labeled bacteria were analyzed by flow cytometry using FACSCalibur (BD Biosciences, Franklin Lakes, NJ, USA).

### Flow Cytometry

Phenotyping of resting and activated moDCs was performed by flow cytometry using anti-human CD1d-phycoerythrin (PE), CD103-FITC, HLA-DQ-FITC, PD-L1-PE (BD Biosciences, Franklin Lakes, NJ, USA), CD1a-allophycocyanin (APC), CD40-FITC (BioLegend, San Diego, CA, USA), CX_3_CR1-PE, CD80-FITC, CD83-FITC, CD86-PE, DC-SIGN-FITC, CCR7-PE, CD14-PE (R&D Systems, Minneapolis, MN, USA), B7RP1 (ICOSL)-PE (EBiosciences, Santa Clara, CA, USA), and isotype-matched control antibodies. The ratio of regulatory T-lymphocytes was measured by flow cytometry using anti-human CD25-PE (BD Pharmingen), CD4-FITC (BioLegend), FoxP3-APC (R&D Systems), and anti-IL-10-AlexaFluor488 (BioLegend). The viability of moDCs was determined with 2 μg/ml 7-amino-actinomycin D (LKT Laboratories Inc., St. Paul, MN, USA) dye followed by a 24-h activation period with live bacteria or LPS. Fluorescence intensities were measured by FACSCalibur (BD Biosciences), and data were analyzed by the FlowJo software (Tree Star, Ashland, OR, USA).

### RNA Isolation, cDNA Synthesis, and Real-time Quantitative PCR

Briefly, total RNA was isolated by TriReagent (Molecular Research Centre, Inc., Cincinnati, OH, USA). Total RNA (1 μg) was reverse-transcribed using High-Capacity cDNA Reverse Transcription Kit (Thermo Fischer Scientific). Gene-specific TaqMan assays (Thermo Fischer Scientific) were used to perform qPCR in a final volume of 12.5 μl in triplicates using DreamTaq DNA polymerase and ABI StepOnePlus real-time PCR instrument. Amplification of h36B4 was used as normalizing controls using specific primers and probe (Integrated DNA Technologies, Coralville, IA, USA). Cycle threshold values were determined using the StepOne Software, version 2.1 (Thermo Fischer Scientific). The sequences of the primers and probes are available upon request.

### Measurement of Cytokine Concentration

Culture supernatants of moDCs were harvested 24 h after moDC activation, and the concentration of TNF-α, IL-1β, IL-6, IL-10, IL-12(p70), IL-23(p19) cytokines, and chemokine CXCL8 was measured using OptEIA kits (BD Biosciences) following the manufacturer’s instructions.

### Stimulation of moDCs to Measure T-Lymphocyte Polarization

To analyze the polarized effector T cells, immature and activated moDCs were washed and co-cultured with peripheral blood lymphocytes (PBLs) for 4 days in AIM-V medium at a moDC:T-cell ratio of 1:20. The T cells were analyzed for IFNγ and IL-17 secretion by the avidin-horseradish peroxidase (HRP)-based enzyme-linked ImmunoSpot system (NatuTec GmbH, Frankfurt am Main, Germany). The co-cultures containing resting moDCs and T-cells as well as T-cells alone served as negative controls. To detect IL-17 secretion, the plates were coated with 0.5 μg/ml mouse anti-hCD3 antibody (BD Biosciences). The plates were analyzed by using the ImmunoScan plate reader (Cell Technology Limited, Shaker Heights, OH, USA). To detect regulatory T-lymphocytes, activated and resting moDCs were washed and co-cultured with PBL or naïve CD4^+^ T-lymphocytes for 6 days in serum-free AIM-V medium at a moDC:T-cell ratio of 1:10. On day 6, cells were harvested, permeabilized, and fixed with Citofix/Cytoperm intracellular staining kit (BD Biosciences). The ratio of CD4^+^CD25^+^FoxP3^+^ T cells was measured by flow cytometry. To detect the presence of intracellular IL-10, T cells were treated on day 6 with Golgi-Stop™ containing monensin (BD Biosciences) for 6 h followed by the surface CD25, CD4, and intracellular FoxP3 and IL-10 staining of cells. Naïve CD4^+^ T-lymphocytes were isolated by the Naïve CD4^+^ T Cell Isolation Kit II, human (Miltenyi Biotec).

### Stimulation of moDCs to Measure iNKT Cell Expansion

Two-day moDCs were co-incubated with live bacteria, LPS, or 100 ng/ml α-galactosylceramide (α-GalCer, KRN7000, Funakoshi, Tokyo, Japan) for 24 h in AIM-V medium. Activated and resting moDCs were washed and co-cultured with PBL for 5 days in AIM-V medium at a moDC:T cell ratio of 1:10 in 24-well plates in AIM-V medium. On day 5, cells were labeled with anti-human CD3-PECy5, T cell receptor (TCR) Vα24-FITC, TCR Vβ11-PE monoclonal antibodies (Beckman Coulter, Brea, CA, USA), and the double-positive iNKT population was monitored by flow cytometry using FACSCalibur.

### Western Blotting

Cells were lysed in Laemmli buffer, and the protein extracts were tested by antibody specific for IRF4 diluted to 1:1,000; secondary antibodies were used at 1:10,000. Anti-rabbit antibody, conjugated to HRP (GE Healthcare Life Sciences, Little Chalfont Buckinghamshire, UK), was used as a secondary antibody. The SuperSignal ECL system was used for probing target proteins (Thermo Fischer Scientific). After the membranes had been probed for the target protein, they were stripped and re-probed for β-actin.

### Statistical Analysis

Student’s unpaired two-tailed *t*-test or ANOVA followed by Bonferroni’s multiple comparison tests were used as indicated in the relevant experiments. In case of significantly different variances (*P* < 0.05) between the two sets of samples, the Welch’s correction was applied in the *t*-test. The results were expressed as mean + SD. All analyses were performed by using the GraphPad Prism software, version 6.0 (GraphPad Software Inc., La Jolla, CA, USA). Differences were considered to be statistically significant at *P* < 0.05. Significance was indicated as *P < 0.05; **P < 0.01; ***P < 0.005; and ****P < 0.0001.

## Results

### The Expression Profile of Master Transcription Factors and the Cell Surface Expression of CD1 Glycoprotein Receptors Differ in Human moDCs

**Figure 1:**
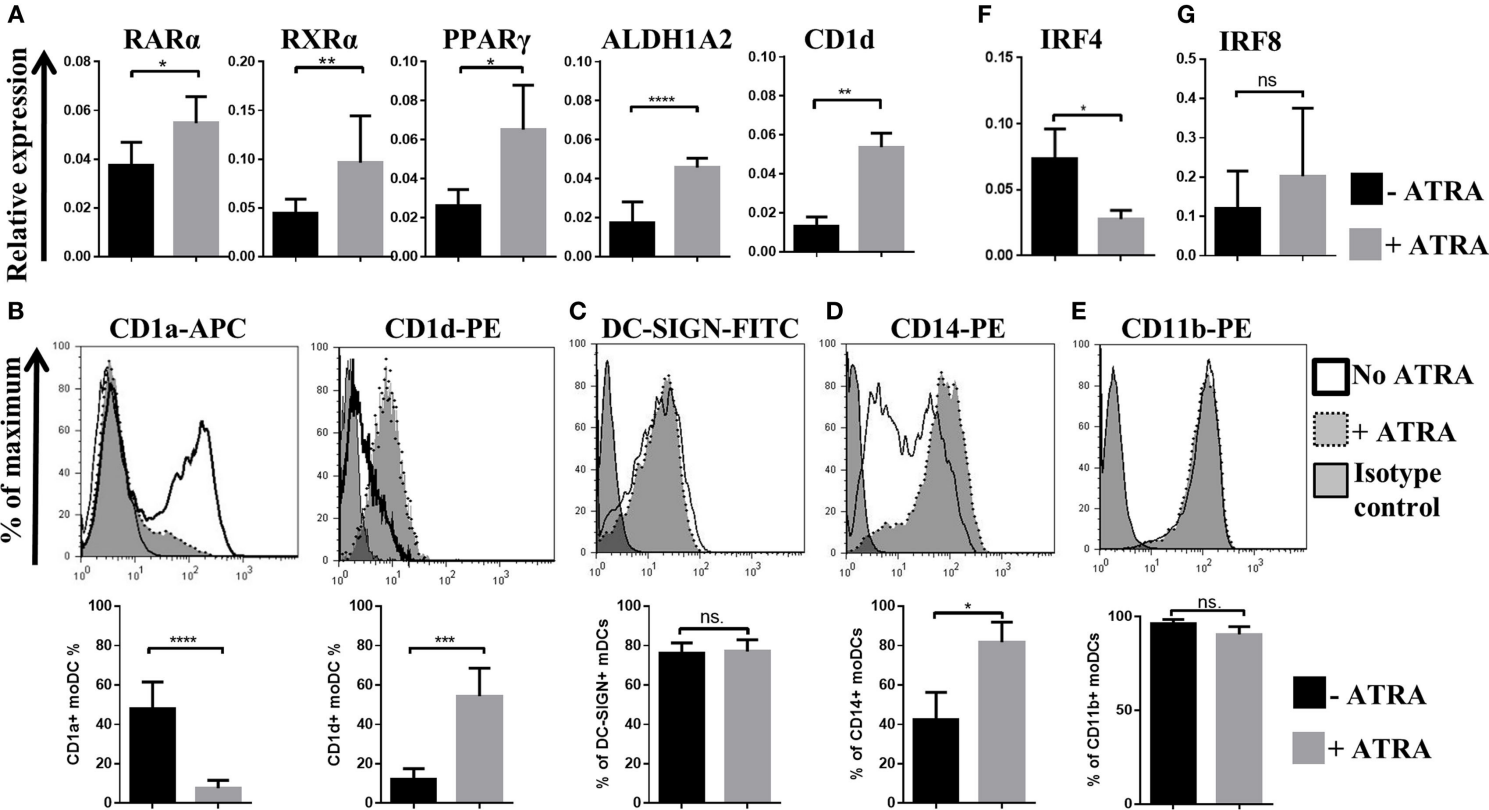
**The effects of all-*trans* retinoic acid (ATRA) on human monocyte-derived dentritic cell (moDC) differentiation**. Two-day moDCs were differentiated in the absence or presence of 1 nM ATRA. The relative gene expression levels of retinoic acid receptor alpha (RARα), retinoic X receptor alpha (RXRα), peroxisome proliferator-activated receptor gamma (PPARγ), ALDH1A2, and CD1d **(A)**, interferon regulatory factor (IRF)4 **(F)**, and IRF8 **(G)** were measured by quantitative real-time PCR, and the cell surface expression level of CD1a, CD1d **(B)**, DC-SIGN **(C)**, CD14 **(D)**, and CD11b **(E)** was measured by flow cytometry. Mean values of relative mRNA levels and the ratio of moDCs positive for the measured cell surface proteins were calculated from five independent experiments +SD. Student’s unpaired two-tailed *t*-test was used in the statistical analysis with significance defined as *P < 0.05, **P < 0.01, ***P < 0.001, and ****P < 0.0001.

We found that in the presence of 1 nM ATRA, monocytes generated in the presence of GM-CSF and IL-4 induced the differentiation of monocytes to moDCs within 2 days accompanied by the increasing expression levels of genes encoding the nuclear hormone receptor RXRα as well as its dimerization partners RARα and PPARγ in line with the aldehyde dehydrogenase-1 family member A2 (ALDH1A2)/retinaldehyde-dehydrogenase 2 (RALDH2) gene (Figure 1A) playing role in the regulation of retinoic acid production in moDCs. In the absence of ATRA, the CD1d gene was expressed in moDCs at low levels, but the CD1d gene transcripts and the cell surface expression of the translated protein was upregulated, while in ATRA-conditioned moDCs, the cell surface expression of CD1a decreased (Figure 1B). Moreover, on days 2 and 3, the differentiation of moDCs could be re-programmed to induce CD1d but inhibited CD1a expression, respectively (data not shown). Importantly, the cell surface expression of the DC-specific intercellular adhesion molecule-3-grabbing non-integrin (DC-SIGN) remained constant at these conditions (Figure 1C), while ATRA maintained the expression level of CD14 (Figure 1D) suggesting a decelerated differentiation phase of moDCs.

Dendritic cells can also be classified according to the expression levels of the transcription factors guiding both DC differentiation and re-programming (39, 40). Murine models suggested that CD11b^+^ bone marrow-derived DCs cultured in the presence of GM-CSF and IL-4 express IRF4 and regulate the cell surface expression of the major histocompatibility gene complex II (MHC class II), while IRF4 increases the antigen-presenting capacity of moDCs resulting in potent T helper cell priming (41). In this human *in vitro* model system, we also found that moDCs express CD11b independent on the presence of ATRA (Figure 1E). Interestingly, ATRA was able to downmodulate the gene expression levels of IRF4 (Figure 1F) while upregulated the cell surface expression of CD103 (Figures 3D,E). Importantly, the relative mRNA level of interferon regulatory factor (IRF)8, responsible for regulating CD103 protein expression in DCs (41), remained unaffected by ATRA (Figure 1G). Collectively, these results demonstrate that nanomolar concentration of ATRA has the potential to modify the moDC differentiation program in a coordinated manner leading to increased mRNA levels of PPARγ, retinoid receptors, ALDH1A2, and CD1d, while the expression of CD1a and IRF4 remained inhibited. Based on this finding, we were able to identify two separate moDC subsets exhibiting distinct phenotypic characteristics based on the expression patterns of CD1 and CD103 proteins and transcription factors. The ATRA-primed CD1a^−^CD103^+^CD1d^+^ cells are the RARα^hi^IRF4^lo^ subpopulation, and in contrast to this combination, the CD1a^+/−^CD103^−^CD1d^−^ cells are identified as a resting RARα^lo^IRF4^hi^ cell population.

### Stimulation of RARα^lo^IRF4^hi^ moDCs by Non-Pathogenic Commensal Bacteria Polarize Effector T-Lymphocytes Differently as Compared to RARα^hi^IRF4^lo^ Cells

**Figure 2:**
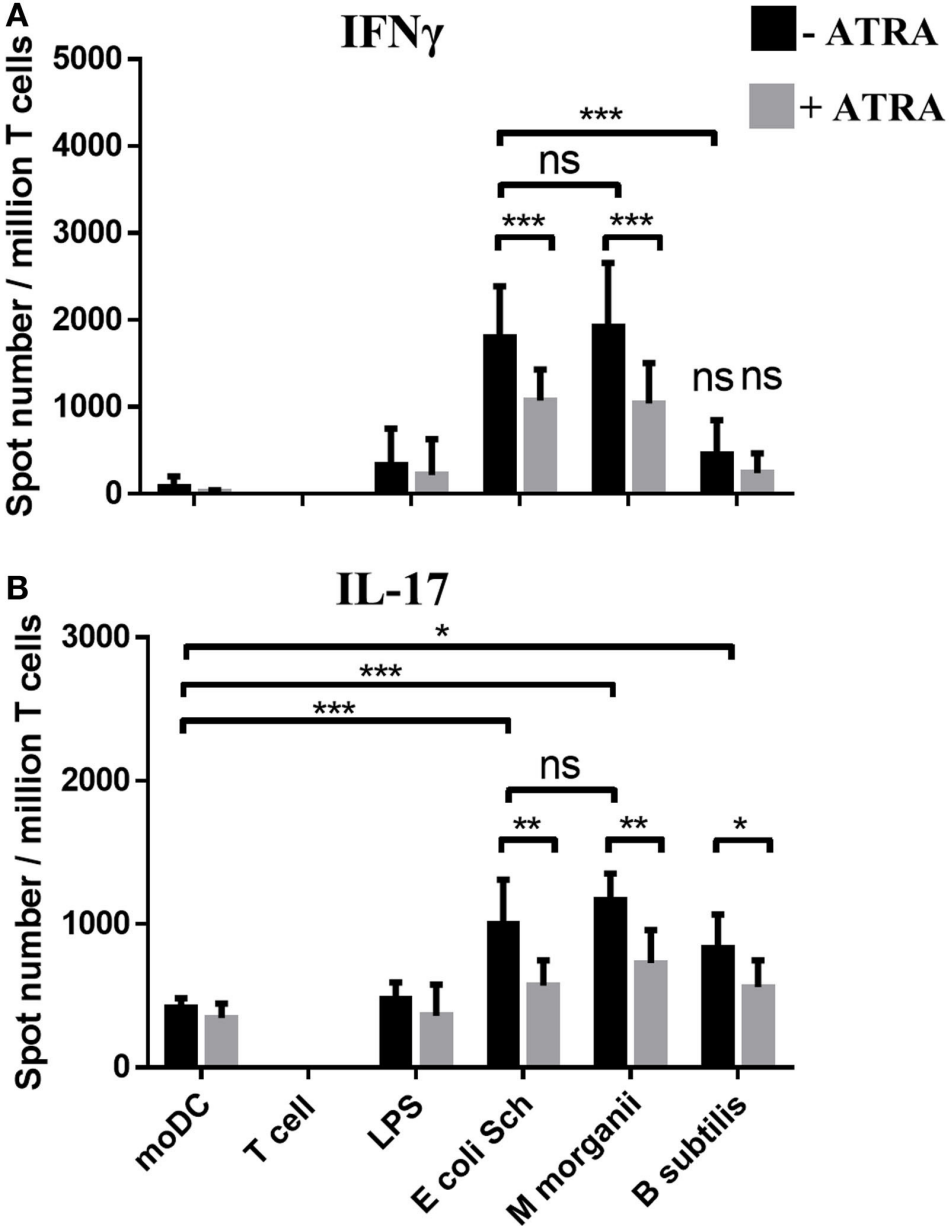
**Monitoring monocyte-derived dentritic cell (moDC)-mediated T-lymphocyte polarization induced by commensal stimuli**. The T cell polarizing capacity of moDCs was monitored in moDC stimulated with *Escherichia coli Schaedler, Morganella Morganii*, and *Bacillus subtilis* or lipopolysaccharide (LPS) followed by co-culturing the cells with freshly isolated autologous T cells for 4 days. The number of cytokine producing T-lymphocytes induced by LPS or by moDCs exposed to commensal bacteria was measured by interferon gamma (IFNγ) **(A)** and interleukin (IL)-17 **(B)** enzyme-linked ImmunoSpot assays. T corresponds to T cells cultured without dendritic cells as negative control. The mean value of spot numbers was calculated from five independent experiments +SD. ANOVA followed by Bonferroni’s multiple comparison tests was used in the statistical analysis with significance defined as *P < 0.05, **P < 0.01, and ***P < 0.001.

Besides the novel finding showing that the outcome of the inflammatory response of DCs to engulfed commensal bacteria is determined by the unique characteristics of the tested microbes (42), we were able to follow-up the immunomodulatory properties of a given microbe though monitoring the activation state and the direction of cell polarization of moDC-mediated autologous T-lymphocytes. In this experimental setting, moDCs were activated by live *E. coli Schaedler* or *M. morganii* both of them being capable to increase the number of IFNγ-producing T-lymphocytes (Figure 2A). By contrast, the Th17 response could be activated by all of the tested species (Figure 2B). In addition, ATRA-conditioned moDCs exhibited a completely different T-lymphocyte stimulatory potential as compared to moDCs manipulated in the absence of ATRA. In this case, the number of IFNγ-secreting T cells was decreased, while that of the Th17 cells remained undetectable in the moDC–T cell co-cultures. Taken the individual features of commensal bacteria, the RARα^lo^IRF4^hi^ moDCs could be activated by both *E. coli Schaedler* and *M. morganii* leading to the differentiation of CD4^+^CD25^+^FoxP3^+^ regulatory T-lymphocytes, while the RARα^hi^IRF4^lo^ reduced this effect (Figures S1A,B in Supplementary Material). To confirm this unexpected observation, we validated the existence of the regulatory T-cell population by detecting the level of the IL-10 cytokine derived from CD4^+^CD25^+^FoxP3^+^ T-lymphocytes co-cultured with moDCs upon the prior activation by commensal bacteria (Figure S1C in Supplementary Material). Based on these results, we were able to identify two moDC populations, which respond to gut commensal species differently, but in a strain- and ATRA-dependent manner. To get further insight how microbiota species guide immune responses of distinct characteristics, we sought to analyze the impact of selected bacterial strains driving the differentiation and functional activities of moDCs by using various experimental approaches.

### The Commensal *E. coli Schaedler* and the Probiotic *B. subtilis* Modulate the Cell Surface Expression of CD1, CX_3_CR1, and CD103 Proteins in an ATRA-Dependent Manner

**Figure 3:**
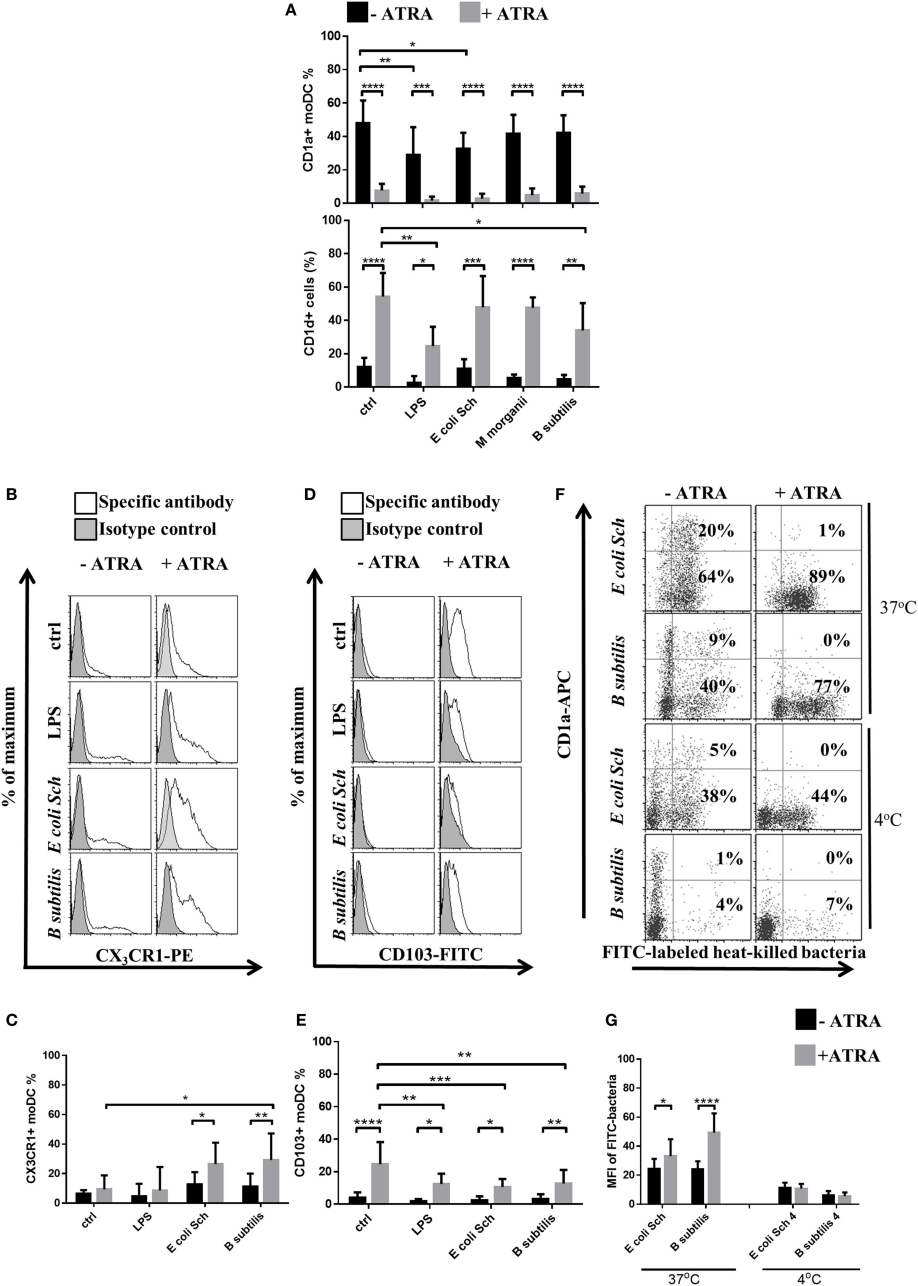
**All-*trans* retinoic acid (ATRA) shifts the cell surface expression pattern of CD1, gut-related receptors, and the phagocytic capacity of monocyte-derived dentritic cells (moDCs) in an ATRA and commensal strain-dependent manner**. Human moDCs were differentiated in the presence of granulocyte-macrophage colony-stimulating factor and interleukin-4 with or without 1 nM ATRA for 2 days. The surface expression level of CD1a and CD1d was measured on resting cells and moDCs activated with live commensal bacteria for 24 h **(A)** by flow cytometry. Histogram overlays show results derived from 1 representative donor of 10. The cell surface expression level of the mucosa-related CX_3_CR1 **(B,C)** and CD103 **(D,E)** was measured by flow cytometry followed by a 24-h activation period with live commensal bacteria or lipopolysaccharide (LPS) served as a positive control. Mean values showing the ratio of moDCs positive for the measured surface protein were calculated from five independent experiments +SD. To monitor the phagocytic capacity of moDCs, on day 2, moDCs were co-cultured with heat-inactivated and fluorescein-isothiocyanate (FITC)-labeled bacteria at 37°C or at 4°C for 3 h at a moDC:bacteria ratio of 1:20. **(F,G)** Dot plots show one of four independent experiments. The ratio of moDC positive for heat-inactivated and FITC-labeled bacteria was measured by flow cytometry. The number of moDCs carrying FITC-labeled bacteria was calculated from four independent experiments +SD. ANOVA followed by Bonferroni’s multiple comparison tests was used in the statistical analysis with significance defined as *P < 0.05, **P < 0.01, ***P < 0.001, and ****P < 0.0001.

To test how gut microbiota strains may act on human moDC differentiation at *in vitro* culture conditions mimicking the intestinal milieu, the cells were exposed to stimulatory signals such as LPS and selected live commensal bacteria. At this experimental setting, exclusively *E. coli Schaedler* was capable to reduce the ratio of CD1a^+^ moDCs indicating the potential of this commensal bacterium to reduce CD1a expression selectively, but it had no effect on CD1d expression (Figure 3A), even though the viability of moDCs remained intact as compared to the immature cells (Figure S2 in Supplementary Material). Interestingly, *B. subtilis* exerted an opposing effect on the cell surface expression pattern of CD1 proteins, and LPS reduced the levels of both CD1d and CD1a in moDCs, while *M. morganii* had no effect on the cell surface expression level of CD1 proteins. These results indicated that lipid antigen presentation by moDCs *via* CD1a and CD1d proteins is regulated by both ATRA and the gut microbiota in a species-specific manner.

Using the *in vitro* system, we established the live commensal bacteria were able to upregulate the cell surface expression of CX_3_CR1 within 24 h (Figures 3B,C) but had no effect on CD103 expression in the absence of ATRA (Figures 3D,E). Moreover, ATRA-conditioned moDCs downregulated the cell surface expression of CD103, but stimulation by commensal bacteria upregulated the CX_3_CR1 receptor. These data altogether confirmed that in the presence of live commensal bacteria, ATRA drives the differentiation of moDCs leading to either synergistic or inhibitory directions, thus modulating the cell surface expression pattern of CD1 and that of the gut-tropic proteins.

### The Phagocytic Capacity of moDCs Depends on the Individual Characteristics of the Tested Bacteria and on Actual Environmental Cues

The very first steps of moDC activation and the induction of antigen-induced immune responses are assisted by the phagocytic potential and the standby physiological activities of moDCs (42). These events can be further modulated by the unique characteristics of the internalized corpuscular antigens as well as by the cell surface receptor repertoire of the given cell. To assess the phagocytic potential of the previously identified moDC populations, we established an *in vitro* phagocytosis assay in which the FITC-labeled heat-inactivated bacteria were exposed to 37°C for 3 h, or were kept at 4°C as control (Figures 3F,G). As expected, the engulfment of commensal bacteria could be enhanced significantly and was found to be mediated by the RARα^hi^IRF4^lo^ moDC population. When the moDCs were co-incubated with FITC-labeled bacteria at 4°C, background fluorescence intensities varied remarkably indicating differences in the individual functional characteristics of the tested commensal bacteria upon penetrating through the moDC membrane. These results altogether confirmed that in the presence of gut-derived microbial stimuli ATRA supports the differentiation of phagocytic CD1a^−^CD1d^+^ moDCs, while the expression of the gut-tropic protein CD103 is partially downmodulated. It was also observed that in the absence of ATRA, the gated CD1a^−^ and CD1a^+^ moDC fractions engulfed the tested bacteria with similar activities as the CD1a^+^ cells (data not shown). Consequently, the median fluorescence intensity values within the gated moDC populations of the FITC-labeled bacteria remained similar demonstrating that the efficacy of moDC-mediated phagocytosis depends on both the unique features and the species of the engulfed bacteria, and this effector mechanism can be further enhanced by ATRA.

### Activation of RARα^hi^IRF4^lo^ moDCs by Commensal Bacteria Provokes Exacerbated Inflammation as Compared to RARα^lo^IRF4^hi^ moDCs

**Figure 4:**
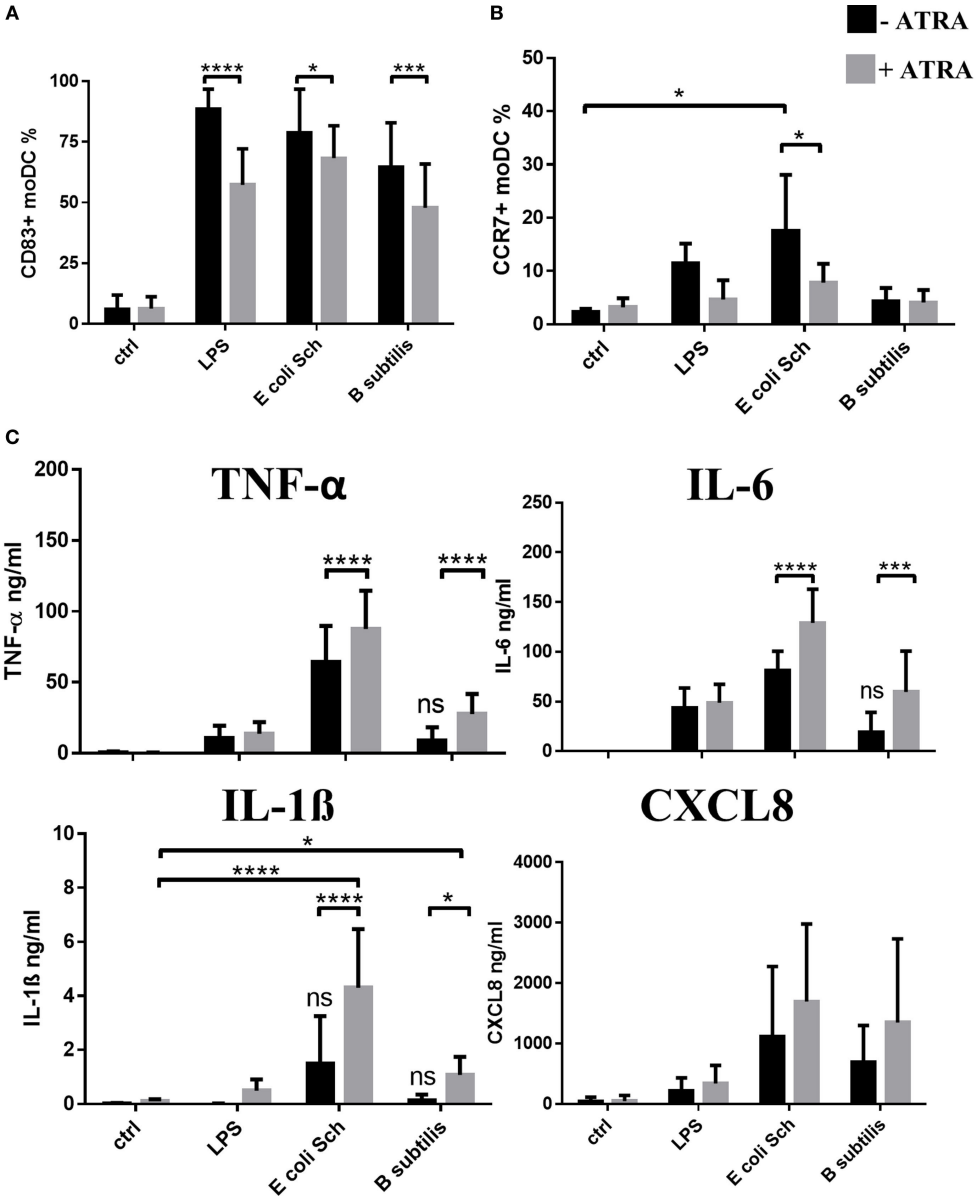
**Characteristics of the inflammatory and migratory potential of monocyte-derived dentritic cell (moDC) populations induced by commensal bacteria**. Two-day moDCs were co-incubated with live commensal strains or with 250 ng/ml lipopolysaccharide (LPS) used as control for 24 h. Expression of the moDC-associated activation marker CD83 **(A)** and CCR7 **(B)** was measured by flow cytometry. Mean values were calculated from five to seven independent experiments +SD. The concentration of TNF-α, interleukin (IL)-1β, IL-6 pro-inflammatory cytokines, and the chemokine CXCL8 **(C)** was measured by ELISA followed by a 24-h activation of moDC in five independent experiments. Mean values +SD are shown. ANOVA followed by Bonferroni’s multiple comparison tests was used in the statistical analysis with significance defined as *P < 0.05, ***P < 0.001, and ****P < 0.0001.

Next, we continued to monitor the inflammatory potential of the selected commensals. Exposure of moDCs to live commensal bacteria such as *E. coli Schaedler* and *B. subtilis* or LPS for 24 h was found to increase the cell surface expression of CD83, while ATRA could downmodulate this response significantly (Figure 4A). The cell surface expression of the chemokine receptor CCR7, playing an essential role in driving DC migration to reach the secondary lymphoid organs, could also be induced in the presence of LPS or *E. coli Schaedler*, but the expression level of CCR7 remained inhibited in ATRA-treated moDC (Figure 4B). In line with these results showing the potential of microbial components to generate mature moDCs, we detected the species-specific production of inflammatory cytokines including TNF-α, IL-1β, IL-6, and CXCL8 chemokine (Figure 4C). Furthermore, *B. subtilis* was found to induce negligible pro-inflammatory cytokine production as compared to Gram-negative *E. coli Schaedler*, but the effects of *B. subtilis* could be boosted significantly upon ATRA treatment confirmed by the increased secretion of TNF-α, IL-1β, and IL-6. We also observed that *M. morganii* induced the expression of a similar panel of moDC-derived inflammatory cytokines as compared to that of *E. coli Schaedler* (data not shown).

These results collectively indicate that *E. coli Schaedler* and *B. subtilis* harbor individual moDC-provoking potential, while ATRA can boost the production of pro-inflammatory mediators. In contrast to this finding, the expression level of CCR7 becomes downmodulated presumably associated with its decreased migratory potential guided by the RARα^hi^IRF4^lo^ moDC population. Based on these results, we conclude that *E. coli Schaedler* acts as a potent inducer of inflammatory responses in moDCs accompanied by the production of TNF-α, IL-1β, and IL-6, while *B. subtilis* is less efficient to trigger TNF-α and/or IL-1β secretion.

### *E. coli Schaedler* and *B. subtilis* Increase the T-Lymphocyte Stimulatory and Polarizing Capacity of moDCs but ATRA Interferes with This Effect

**Figure 5:**
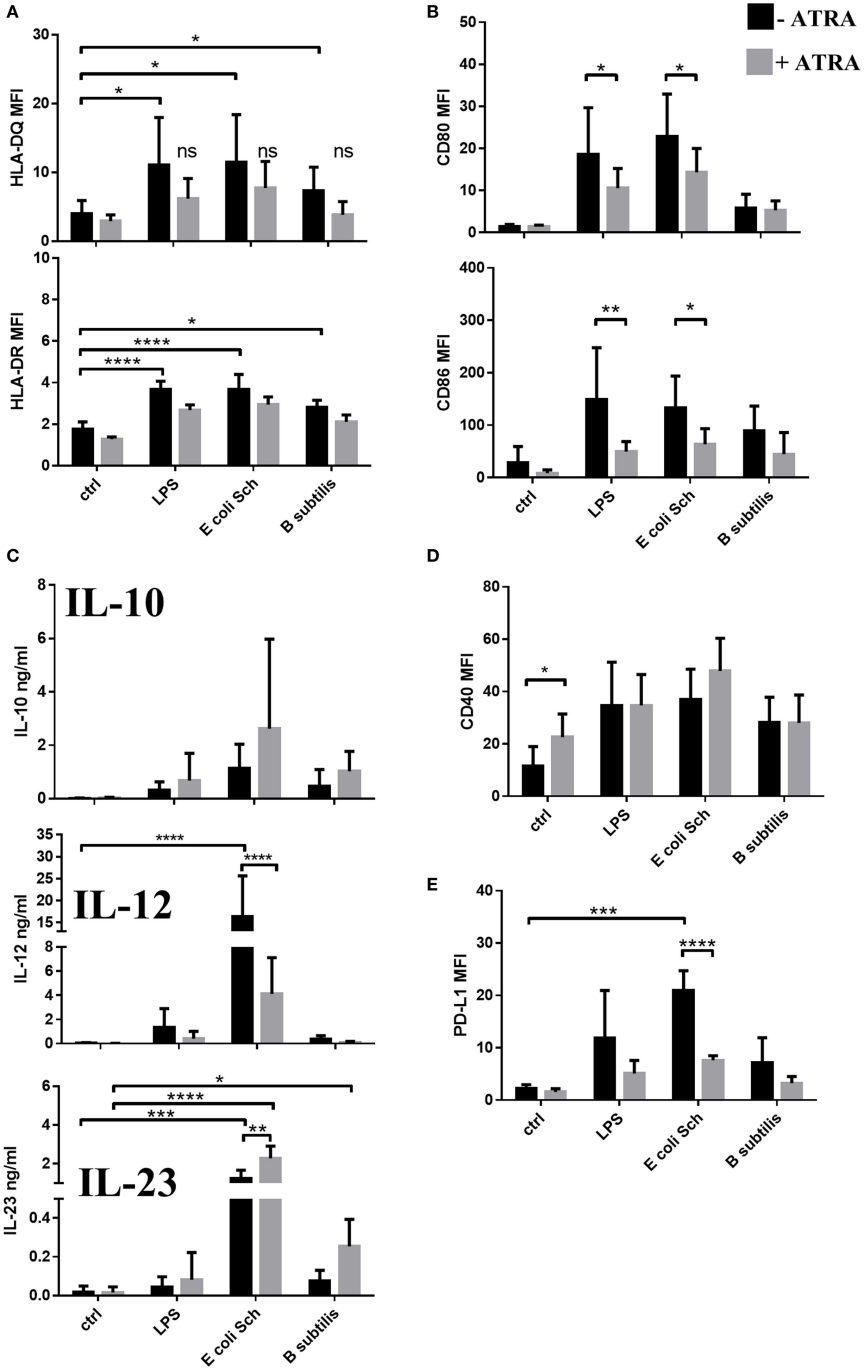
**The T-lymphocyte activating and polarizing capacity of monocyte-derived dentritic cells (moDCs) activated by selected commensal bacteria**. Two-day moDCs were co-incubated with live commensal strains or with 250 ng/ml lipopolysaccharide (LPS) used as control for 24 h. The expression levels of HLA-DQ and HLA-DR **(A)**, the co-stimulatory proteins CD80 and CD86 **(B)**, CD40 **(D)**, and the inhibitory molecule PD-L1 **(E)** was measured by flow cytometry. Mean values of median fluorescence intensities (MFIs) were calculated from five to seven independent experiments +SD. The concentration of interleukin (IL)-12, IL-23, and IL-10 cytokines was measured by ELISA followed by a 24-h activation of moDC and was tested in seven independent experiments **(C)**. Mean values +SD are shown. ANOVA followed by Bonferroni’s multiple comparison tests was used in the statistical analysis with significance defined as *P < 0.05, **P < 0.01, ***P < 0.001, and ****P < 0.0001.

The first signal for Th cell activation derives from the interaction of the TCR with MHC class II–peptide complexes presented by antigen-presenting proteins such as HLA-DQ and HLA-DR inducible by LPS or by the selected microbiota strains (Figure 5A). When moDCs were exposed to LPS or to commensal bacteria, the cell surface expression of the CD80 and CD86 co-stimulatory molecules was increased (Figure 5B). In such an experimental system, the secretion of the regulatory cytokine IL-10 was independent on ATRA in case of moDC activation by bacteria. More importantly, the secretion level of the Th1 polarizing cytokine IL-12 was decreased, while that of the IL-23 cytokine was enhanced significantly in the RARα^hi^IRF4^lo^ moDC population (Figure 5C). Interestingly, *B. subtilis* was unable to induce IL-23 secretion and the level of IL-12 also remained lower than the effect provoked by moDCs in the presence of the Gram-negative commensal bacterium *E. coli Schaedler*.

Considering that the differentiation of T-lymphocytes is regulated by both co-stimulatory and inhibitory signals, the cell surface expression of known co-stimulators of T-lymphocytes were also monitored. The results revealed that the cell surface expression of the co-stimulatory molecule CD40 could be induced by LPS and also by the two commensal strains, and this effect could be slightly enhanced in PPARγ^hi^IRF4^low^ moDCs upon activation by *E. coli Schaedler* (Figure 5D). The induction of the effector T cell inhibitor PD-L1 could also be achieved if moDCs were stimulated by *E. coli Schaedler* (Figure 5E), in contrast to *B. subtilis* or LPS with no such effects. These data altogether suggest that both LPS and gut-associated commensal bacteria can induce the cell surface expression of T cell co-stimulatory and inhibitory molecules on the moDC cell surface in a strain-dependent manner, while ATRA-activated moDCs exhibit impaired cell surface expression of MHC class II, co-stimulatory, and inhibitory cell surface proteins.

### Limited Commensal-Induced Effector Responses Mediated by RARα^hi^IRF4^lo^ moDCs Are Associated with Augmented Inflammation That Can Be Rescued by the Selective Inhibition of RARα

**Figure 6:**
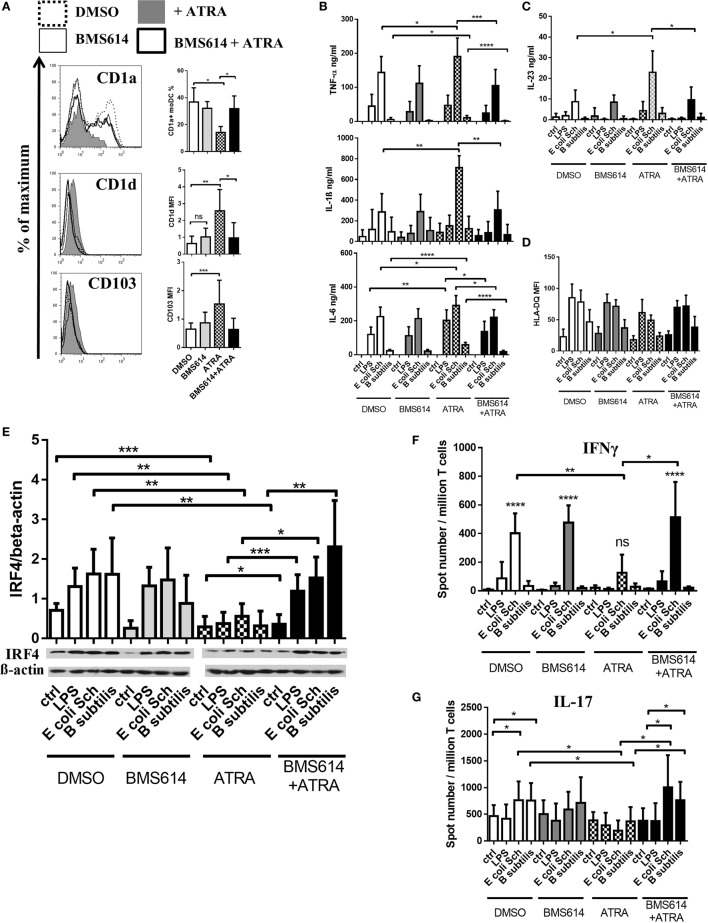
**The selective inhibition of retinoic acid receptor alpha (RARα) prevents the all-*trans* retinoic acid (ATRA)-induced signature of microbiota-generated immune responses mediated by monocyte-derived dentritic cells (moDCs)**. To analyze how ATRA acts on the moDC-mediated immune response against microbiota species, the cells were treated with the RARα antagonist BMS614 prior to treating the cell culture medium with ATRA. The cell surface expression level of CD1a, CD1d, and CD103 was measured by flow cytometry in 2-day moDCs **(A)**. The concentration of TNF-α, interleukin (IL)-6, and IL-1β **(B)** and IL-23 **(C)** was measured by ELISA followed by a 24-h activation of moDC performed in seven independent experiments. Mean values +SD are shown. The cell surface expression level of HLA-DQ was measured by flow cytometry followed by a 24-h incubation period with live commensal bacteria. **(D)** Mean values of cells positive for the measured cell surface molecules were calculated from the results of seven independent donors +SD. Analysis of interferon regulatory factor 4 (IRF4) expression in moDCs. **(E)** Two-day moDCs were activated by live commensal bacteria for 24 h, and the relative expression levels of IRF4 protein was measured by Western blotting. Bar graphs show IRF4/β-actin ratios measured after 24 h of stimulation. Mean values of protein densities were calculated from five independent experiments +SD. The T cell polarizing capacity of moDCs was monitored in moDCs activated with the selected commensal strains or with lipopolysaccharide (LPS) followed by co-culturing the cells with autologous T cells. Freshly isolated peripheral blood lymphocytes were co-cultured with autologous moDCs for 4 days. The number of cytokine producing T-lymphocytes, induced by LPS or moDCs exposed to *Escherichia coli Schaedler* and *Bacillus Subtilis*, was measured by interferon gamma (IFNγ) **(F)** and IL-17 **(G)** enzyme-linked ImmunoSpot assays. The mean value of spot numbers was calculated from five independent experiments +SD. Statistical analysis was performed by the Student’s unpaired two-tailed *t*-test with significance defined as *P < 0.05, **P < 0.01, ***P < 0.001, and ****P < 0.0001.

In a next step, we addressed the question how T-lymphocyte stimulation and maturation may modulate moDC responses in the presence of ATRA or commensal bacteria. Taken the fact that differentiation of moDCs can be modified in the presence of 1 nM ATRA, we also confirmed that the blockade of RARα signaling by a specific antagonist resulted in the prevention of CD1d and CD103 expression, while in the presence of ATRA, the cell surface expression of CD1a remained similar as control cells (Figure 6A). The chemical antagonist of RARα, i.e., BMS614 was unable to increase the cell surface expression level of CD1a on the cell surface showing that a minimal concentration of endogenous ATRA is presented by moDCs.

In a further step, we also demonstrated that the enhanced secretion of the pro-inflammatory cytokines (Figure 6B) and IL-23 (Figure 6C) induced by commensal bacteria could be ameliorated by the prior blockade of RARα. Moreover, the reduced antigen-presenting capacity of the ATRA-conditioned moDCs could be restored by the inhibition of RARα (Figure 6D).

Considering that the IRF4 transcription factor plays a pivotal role in setting the degree of DC-mediated antigen presentation (41), in a final experimental setting, we described for the first time in human moDCs that the protein level of IRF4 could be upregulated by live commensal bacteria and this effect could be decreased in a RARα-dependent manner (Figure 6E). As we expected, the decreased effector T-lymphocyte polarizing capacity of moDCs could be recovered by the selective blockade of RARα leading to strong Th1 (Figure 6F) and Th17 (Figure 6G) responses against the selected microbiota strains. Based on these results, we propose that the differentiation program of moDC initiated by GM-CSF and IL-4 can readily be modulated by ATRA, and this effect is associated specifically to the RARα nuclear receptor. In line with the results showing that ATRA is able to downmodulate the gene expression of IRF4 in both resting and ATRA-conditioned activated moDCs in the presence of commensal bacteria, the cell surface expression of antigen-presenting HLA-DQ molecules is decreased in a RARα-dependent manner.

## Discussion

This study focuses to the interplay of moDCs differentiated *in vitro* and designed to accommodate to various microenvironments having the potential to guide autologous effector T-lymphocyte functional activities. In this context, the polarization and the actual expression patterns of the cell surface molecules exhibiting co-stimulatory and/or inhibitory potential were monitored in the presence and absence of selected members of the gut microbiota exemplified by *E. coli Schaedler, M. morganii*, and *B. subtilis*. Based on our concept, the outcome of moDC differentiation is able to accommodate to unique cellular microenvironments (21, 44) and remains remarkably plastic until the terminal differentiation of the moDCs ensues. In line with this, we also demonstrated that during the very early phase of moDC differentiation, the cells remain programmable at physiologically relevant doses of environmental cues such as in the presence of nanomolar ATRA (45). Importantly, these events can be prevented by the selective ligation of RARα acting through its natural antagonist resulting in a moDC phenotype similar to that of the “gold standard” of moDCs (43) differentiated by GM-CSF and IL-4.

**Figure 7:**
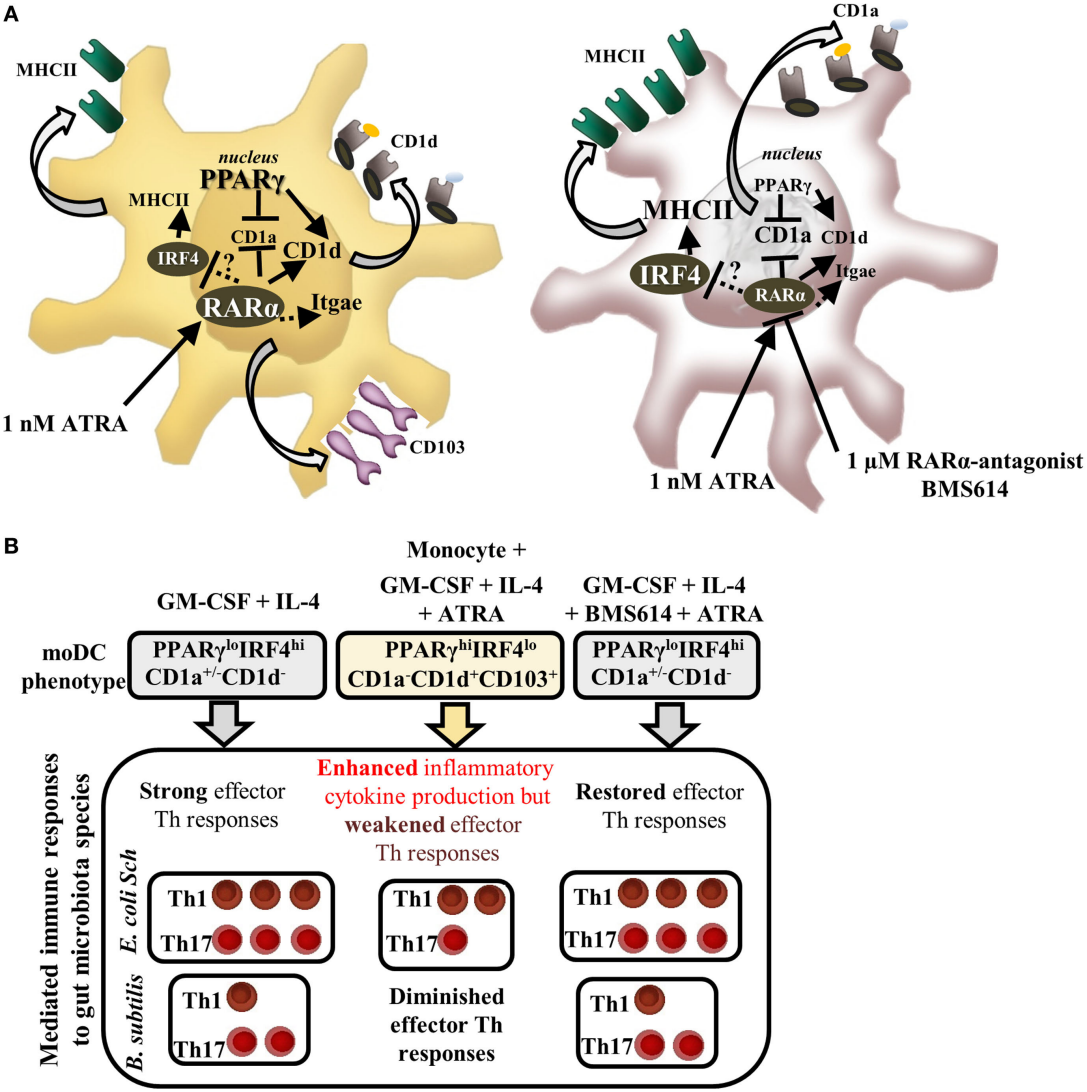
**The role of retinoic acid receptor alpha (RARα) in guiding monocyte-derived dentritic cell (moDC) development and microbiota-induced immune responses**. All-*trans* retinoic acid (ATRA) modifies the differentiation of moDCs that could be prevented by the selective inhibition of RARα. **(A)** In the presence of granulocyte-macrophage colony-stimulating factor (GM-CSF) and interleukin (IL)-4, monocytes differentiate to CD1d^−^CD1a^+^ DCs (43). Peroxisome proliferator-activated receptor gamma (PPARγ) and RARα regulate the gene expression of CD1d and ALDH1A2 both directly and indirectly in human moDCs (20). Interferon regulatory factor 4 (IRF4) mediates the differentiation and antigen-presenting capacity of human moDCs, which is downregulated by the ligation of RARα resulting in decreased mRNA and protein levels of IRF4 together with CD1a. Selected microbiota species provoke different types of immune responses mediated by moDCs. **(B)** *Escherichia coli Schaedler* induces full maturation in moDCs leading to strong inflammatory and microbiota-induced effector helper T (Th) responses, while *Bacillus subtilis* induces inflammation in the absence of IL-12 and IL-23 and provokes decreased effector Th1/Th17 immune responses. ATRA downmodulates the immunogenicity of moDCs resulting in diminished Th1 and undetectable Th17 responses, which effect can be restored in moDCs by the prior inhibition of RARα in moDCs. Solid lines represent known mechanisms; dotted lines indicate unknown molecular interactions.

In a retinoid-rich milieu, moDCs shift the cell surface expression pattern of CD1 proteins, and in resting moDCs, the expression level of CD103 remains inducible supporting the development of a mucosa-related phenotype (46, 47). This observation allowed us to distinguish the characteristics of the expressed cell surface molecules such as CD1 and CD103 on various moDC types. These proteins can be expressed by the CD1a^+/−^CD1d^−^CD103^−^ and the CD1a^−^CD1d^+^CD103^+^ cell populations, respectively (Figure 7A).

We first characterized and compared the expression levels of the contributing transcription factors, including IRF4, PPARγ, and RARα in moDCs. DCs expressing IRF4 were shown to be the less potent inducers of cytotoxic T-lymphocytes as compared to cells expressing IRF8, a DC subset localized to the gut mucosa (39, 48). The results revealed that IRF4^hi^ moDCs can be characterized as immunogenic cells provoking commensal-induced Th1 and Th17 immune responses, but this pattern could be reduced in case the T cells were primed with microbiota-stimulated RARα^hi^PPARγ^hi^IRF4^lo^ moDCs supporting the notion that these cells remain highly inflammatory, lose their potential to activate autologous effector T helper cells, and also lack molecular interactions, which may play role in preventing effector T cell responses induced by commensal bacteria (Figure 7B). This observation is further supported by previous studies showing that the increased expression level and activity of PPARγ is associated with CD1d expression and the development of tolerogenic moDCs (20). Ligation of the CD40 cell surface molecule enhances the inflammatory potential of DCs (49) and the resting moDCs concomitantly conditioned with ATRA upregulate the cell surface expression of CD40, which can be further increased by *E. coli Schaedler* as compared to moDCs differentiated in the absence of ATRA. This observation is also confirmed by the concept that resting DCs express high levels of CD40 on the cell surface representing a semi-activated DC population with tolerogenic features (50, 51).

It has also been demonstrated that in the presence of heat-killed *E. coli Schaedler* and *B. subtilis* bacteria, the phagocytic capacity of moDCs could be facilitated by ATRA, similar to a previous work showing increased PPARγ activity in moDCs upon internalizing corpuscular antigens more efficiently than moDCs with low PPARγ activity (52). In addition to these findings, we also demonstrated that the stimulation of moDC with selected commensal bacteria resulted in moDCs expressing CX_3_CR1 supported by ATRA and showing a phenotype similar to that of the CD11b^+^CX_3_CR1^+^CD103^−^ mononuclear mucosal phagocytes of myeloid origin. Moreover, in the presence of selected bacterial strains, the ATRA-primed moDCs induced the secretion of pro-inflammatory cytokines including TNF-α, IL-1β, IL-6, and IL-23 at high levels. Considering that these inflammatory cytokines play central role in the maintenance and/or disruption of mucosal integrity, exemplified by secreted IL-23 of both DC and macrophage origin. These regulatory circuits may serve as double-edged swords in the maintenance of balance in health and disease. The increased level of secreted IL-23 could directly be associated with several chronic inflammatory diseases including IBD (53). However, the presence of microbiota provide signals for both CX_3_CR1^+^ inflammatory cells and CD11b^+^CD103^+^ DCs in the *lamina propria* to produce IL-23 and induce IL-22 secretion by innate lymphoid cells, thus playing a critical role in promoting mucosal healing in colitis (37, 54). Pro-inflammatory *lamina propria*-derived TNF-α can also exacerbate colitis through CX_3_CR1^+^ DCs indicating that this DC subset also plays role in the maintenance of balanced inflammatory and/or standby conditions upon gut homeostasis (32).

In the presence of live bacteria, ATRA boosts the secretion of Th17 polarizing cytokines; however, the polarizing capacity of these moDCs is reduced. This observation is also supported by our previous study showing that moDCs “educated” by the supernatant of ATRA-primed colonic epithelial cells were able to reduce CCR7-dependent cell migration as well as their Th17 polarizing capacity as compared to control moDCs (44). Interestingly, in a murine model, Th17 differentiation was found to be dependent on IRF4 and IL-6 secreted by CD11b^+^CD103^+^ DCs derived from the mesenteric lymph nodes (55). The same group also showed that the human equivalent of these DCs could be identified as the intestinal IRF4 protein expressing CD103^+^SIRPα^hi^ DCs.

Based on the known regulatory functions of DCs, this study demonstrates that the selected commensal bacteria also secrete IL-10, an inhibitory cytokine acting independently on the bacterial species. At our experimental conditions, the cell surface expression of PD-L1 protein became upregulated in a bacterial strain-dependent manner, which could be demonstrated also in the ATRA-primed moDCs, even though its expression level was significantly lower as compared to the respective ATRA free moDC counterpart. In addition to these results, the secretion of IL-12 cytokine with known inflammatory properties was downmodulated by ATRA as shown before by others (56). In contrast to these findings, we demonstrated that ATRA had no effect on IL-10 secretion in moDCs. Collectively, these data indicate that the decreased levels of IL-12, the reduced co-stimulatory and antigen-presenting capacity of RARα^hi^IRF4^lo^ moDCs, together with the production inhibitory IL-10 create a local milieu, which is inefficient to induce potent effector T helper cell responses upon targeting the selected gut microbiota species.

Our results clearly demonstrated that in resting moDCs, ATRA is able to upregulate the relative mRNA levels of RARα, previously confirmed also by others (56). In addition, we can exclude the effects of other RAR isoforms such as RARβ, as it is not expressed and the expression of RARβ could not be induced in moDCs in the presence of ATRA. It has also been shown that the effects of ATRA on the differentiation and the microbiota-induced stimulation of moDCs could be prevented by the selective inhibition of RARα, a transcription factor playing critical role in regulating moDC differentiation and guiding mucosal immune responses. It has also been found that the gut microbiota has an impact on retinoid signaling-mediated immune homeostasis transmitted by microbial metabolites such as short-chain fatty acids (57). Furthermore, retinoid supplementation through diet also acts on the composition of the gut microbiota and on energy metabolism of the host (58). For example, vitamin A deficiency causes perturbations in the gut microbiota by reducing the ratio of *Firmicutes* and *Proteobacteria* on a Myd88- and TRIF-dependent manner (59). It has previously been demonstrated that RA is associated to inflammatory macrophages, as patients with Crohn’s disease exhibit an increased capacity to generate RALDH-derived RA, which is associated with CD14^+^ macrophages derived from the intestinal mucosa, thus maintaining an inflammatory phenotype mediated by RARα (26). This group also showed that clinical samples derived from Crohn’s disease patients involve both CD103^+^ and CD103^−^ DCs with elevated expression levels of the ALDH1A2 gene, which is undetectable in RA-producing macrophages. Retinoids involving ATRA also improves the antitumor immunity in microbiota-induced colorectal cancer, as it increases the efficacy of tumor-specific cytotoxic T-lymphocytes by increasing RARα-mediated MHCI expression in tumor cells (60).

Human moDCs not only provoke antigen-specific immune responses but also induce the activation and expansion of innate lymphoid cells; among them, iNKT cells (20, 61) and also present lipid antigens *via* cell surface CD1 glycolipid receptors. Remarkably, the level of CD1a and CD1d expression can be modified by commensal bacteria to different extents supporting the notion that this effect is not even related to the local lipid/retinoid environment, the activity of PPARγ (21), or the presence of pathogenic microbes (62), but their activities may resemble some microbiota species such as *E. coli Schaedler* and *B. subtilis*. moDCs with increased PPARγ activity also induce the expansion of IFNγ-secreting iNKT cell at high levels as compared to moDCs with low PPARγ activity (52). Surprisingly, we were unable to detect changes in the number of iNKT cells in moDCs stimulated by commensal bacteria, when the activated moDC–T cell cultures were tested. Instead, moDCs generated processed lipid antigens derived from commensal bacteria indicating that these lipids are unable to provide ligands for CD1a or CD1d proteins (Figure S3 in Supplementary Material). However, it was previously reported that bacterial colonization of the murine colon with *E. coli Schaedler* stimulates intestinal epithelial cells and intraepithelial innate lymphoid cells (63) independently, and this effect may play role in the pathogenesis of colitis as demonstrated in adoptive transfer models using SCID mice, which may also operate in patients with IBD.

Collectively, we offer a sensitive *in vitro* assay system appropriate for the comparative analysis of selected individual microbes in the course of collaboration with human phagocytic cells such as primary moDCs, playing essential roles in orchestrating the outcome of immune responses. We also confirmed that the vitamin A derivative ATRA has the potential to drive the differentiation program of moDCs in a RARα-dependent manner and thus confers suppressive signals during gut commensal bacteria-induced effector T-lymphocyte responses in line with enhancing their local inflammatory potential.

The interactions of diet, gut microbiota and the host build up a highly complex network of regulatory circuits to drive the development of both mucosal and systemic immune responses. Preferentially in early childhood, imbalances in food supplementation together with the acquired perturbance of the gut microbiota increase the risk of chronic immune and metabolic disorders; however, how the environmental and genetic factors determine the outcome of such immune failures requires further analysis.

## Ethics Statement

Leukocyte-enriched buffy coats were obtained from healthy blood donors drawn at the Regional Blood Center of the Hungarian National Blood Transfusion Service (Debrecen, Hungary) in accordance with the written approval of the Director of the National Blood Transfusion Service of the University of Debrecen, Faculty of Medicine (Hungary) and from the Regional and Institutional Research Ethical Committee of the University of Debrecen (DEOEC RKEB/IKEB 3855-2013). Written, informed consent was obtained from the blood donors prior blood donation, their data were processed and stored according to the directives of the European Union.

## Author Contributions

KB designed and performed the experiments, analyzed the results, organized the data, and wrote the manuscript. ZV contributed to protein-based experiments. VP contributed to the isolation and cultivation of commensal microbes. NB provided initial experimental idea and revised the manuscript. ER designed the concept, developed the interpretation, and revised the manuscript.

## Conflict of Interest Statement

The authors declare that the research was conducted in the absence of any commercial or financial relationships that could be construed as a potential conflict of interest.

## Supplementary Material

Figure S1 **Regulatory T-lymphocyte polarizing capacity of monocyte-derived dentritic cell (moDC) populations stimulated by *Escherichia coli Schaedler, Morganella morganii*, and *Bacillus subtilis***. To detect the number of regulatory T-lymphocytes, resting and stimulated moDCs were co-cultured with peripheral blood lymphocyte for 6 days. The ratio of CD25^+^FoxP3^+^ Treg cells **(A,B)** and the interleukin (IL)-10-producing Treg cells **(C)** were analyzed by flow cytometry, respectively. Dot plots show one out of five independent experiments. The mean value of Treg cell numbers was calculated from five independent experiments +SD. In the statistical analysis, ANOVA followed by Bonferroni’s multiple comparison tests were used with significance defined as **P* < 0.05.

Figure S2 **Monitoring the viability of monocyte-derived dentritic cells (moDCs) exposed to live commensal bacteria in the absence or presence of all-*trans* retinoic acid (ATRA)**. moDCs were differentiated with or without ATRA for 2 days in serum-free culture medium. On day 2, moDCs were co-incubated with live commensal bacteria for 24 h followed by labeling the cells with 7-amino-actinomycin D (7-AAD) dye. Mean values of moDCs positive for 7-AAD staining were calculated from five independent experiments +SD.

Figure S3 **The invariant natural killer T (iNKT) cell inducing capacity of monocyte-derived dentritic cell (moDC) populations stimulated by *Escherichia coli Schaedler* and *Bacillus subtilis***. To detect the number of iNKT cells, moDCs were stimulated with live bacteria or with lipopolysaccharide (LPS) followed by co-incubation with autologous peripheral blood lymphocyte for 5 days, and the moDC cultures were incubated with the CD1d ligand α-GalCer served as a positive control. The ratio of CD3^+^ cells expressing Vα24Vβ11 T cell receptors was analyzed by flow cytometry. The mean values of iNKT cell numbers were calculated from three independent experiments +SD.
