# Supplementary figures and images for: Gut Microbiota Species Can Provoke both Inflammatory and Tolerogenic Immune Responses in Human Dendritic Cells Mediated by Retinoic Acid Receptor Alpha Ligation
Source: Front Immunol. 2017 Apr 18;8:427. doi: 10.3389/fimmu.2017.00427 (PMC5394128; doi:10.3389/fimmu.2017.00427)

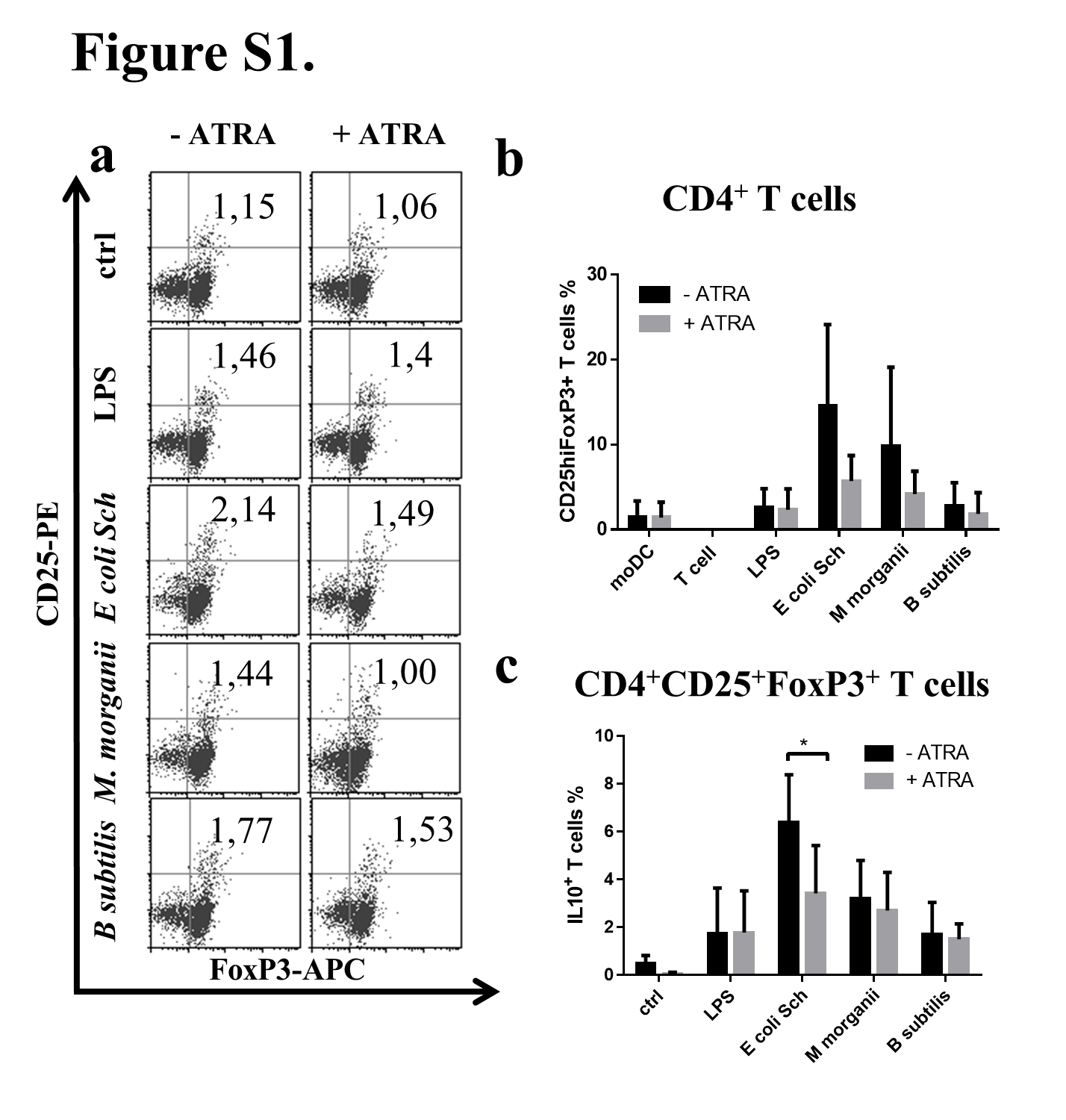

Supplement: Figure S1 — Regulatory T-lymphocyte polarizing capacity of monocyte-derived dentritic cell (moDC) populations stimulated by Escherichia coli Schaedler, Morganella morganii, and Bacillus subtilis. To detect the number of regulatory T-lymphocytes, resting and stimulated moDCs were co-cultured with peripheral blood lymphocyte for 6 days. The ratio of CD25+FoxP3+ Treg cells (A,B) and the interleukin (IL)-10-producing Treg cells (C) were analyzed by flow cytometry, respectively. Dot plots show one out of five independent experiments. The mean value of Treg cell numbers was calculated from five independent experiments +SD. In the statistical analysis, ANOVA followed by Bonferroni’s multiple comparison tests were used with significance defined as *P < 0.05. [file Image_1.tif]

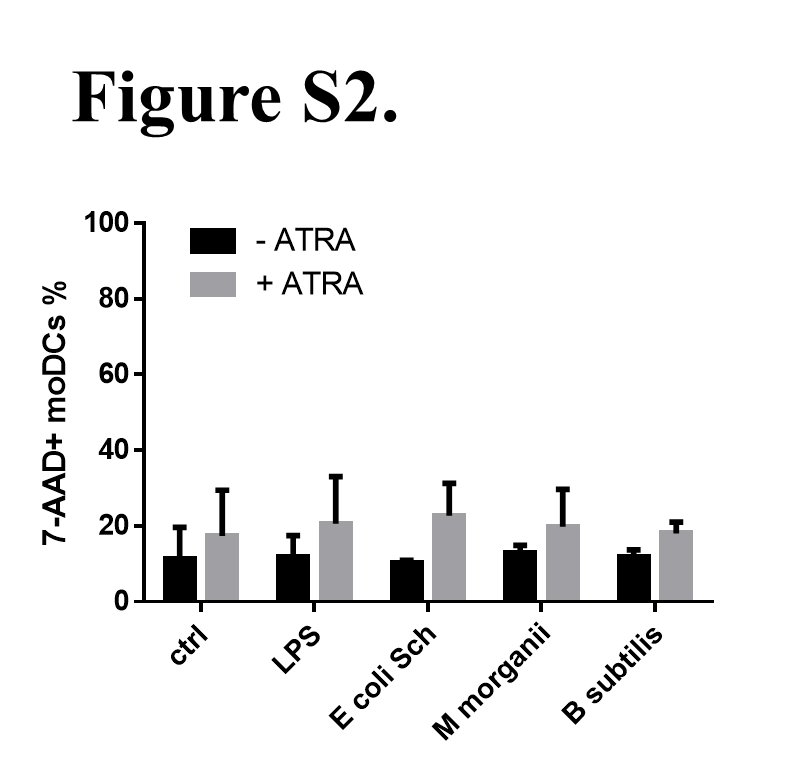

Supplement: Figure S2 — Monitoring the viability of monocyte-derived dentritic cells (moDCs) exposed to live commensal bacteria in the absence or presence of all-trans retinoic acid (ATRA). moDCs were differentiated with or without ATRA for 2 days in serum-free culture medium. On day 2, moDCs were co-incubated with live commensal bacteria for 24 h followed by labeling the cells with 7-amino-actinomycin D (7-AAD) dye. Mean values of moDCs positive for 7-AAD staining were calculated from five independent experiments +SD. [file Image_2.tif]

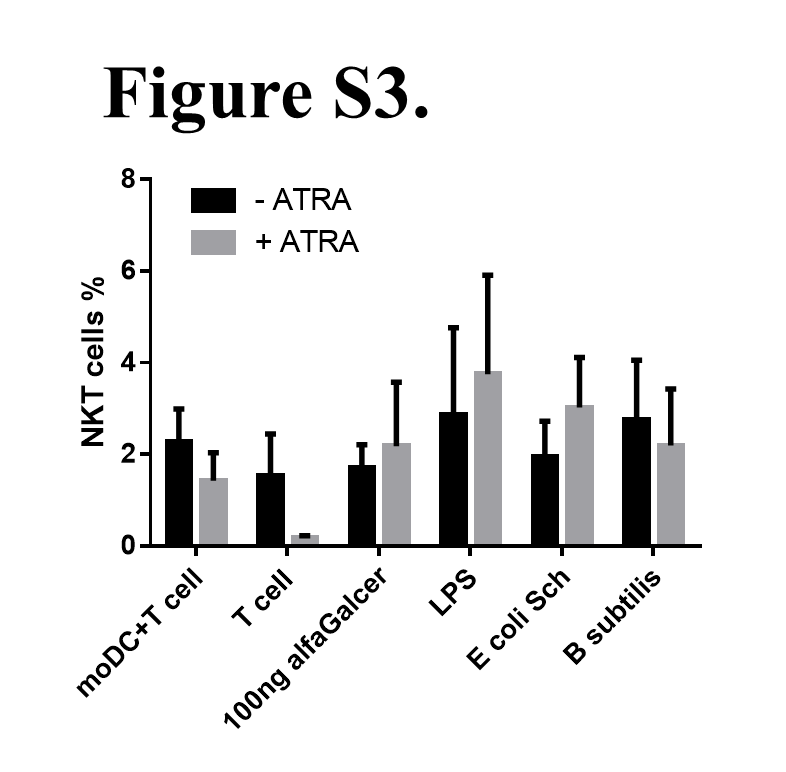

Supplement: Figure S3 — The invariant natural killer T (iNKT) cell inducing capacity of monocyte-derived dentritic cell (moDC) populations stimulated by Escherichia coli Schaedler and Bacillus subtilis. To detect the number of iNKT cells, moDCs were stimulated with live bacteria or with lipopolysaccharide (LPS) followed by co-incubation with autologous peripheral blood lymphocyte for 5 days, and the moDC cultures were incubated with the CD1d ligand α-GalCer served as a positive control. The ratio of CD3+ cells expressing Vα24Vβ11 T cell receptors was analyzed by flow cytometry. The mean values of iNKT cell numbers were calculated from three independent experiments +SD. [file Image_3.tif]
